# Troublesome Weight Loss: A Case Report of Large Gastric Phytobezoar

**DOI:** 10.34172/mejdd.2024.390

**Published:** 2024-07-31

**Authors:** Ali Mohammad Esfandiary Rad, Masoud Mohammad Malekzadeh

**Affiliations:** ^1^Student Research Committee, Neyshabur University of Medical Sciences, Neyshabur, Iran

**Keywords:** Gastric phytobezoar, Coca-Cola, Iran

## Abstract

Gastric bezoar is a very rare clinical condition and hard to treat. Phytobezoars are one of the most common types of bezoars, which happens with the consumption of indigestible fibers. In this report, we presented an elderly man with gastric phytobezoar who presented with peptic ulcer and was treated successfully with proton pump inhibitor (PPI) and Coca-Cola. Surveillance endoscopy showed a completely healed ulcer. It was shown that cautious use of Coca-Cola can be helpful and safe in treating concomitant phytobezoar and gastric ulcers. To the best of our knowledge, this is the first report of phytobezoar from Iran, which was treated with cola.

## Introduction

 Gastrointestinal tract bezoar is a very rare clinical condition and hard to treat. Bezoars are of different types, such as trichobezoar, phytobezoars, pharmacobezoars, etc. Phytobezoars are one of the most common types of bezoars, which happens when materials are consumed that have abundant amounts of indigestible fibers. Phytobezoar is known as a risk factor for peptic ulcer. Here, we present a case of phytobezoar presenting with hematemesis, who had bezoar-induced peptic ulcer, which was successfully treated with Coca-Cola.

## Case Report

 A 79-year-old male patient presented to the emergency department (ED) of 22 Bahman Hospital (Neishabur, Iran) with hematemesis. The patient had used herbs one year ago for a duration of 8-months to reduce weight. The herb was prescribed by a non-specialist, and its exact name and type was unknown. Three months after discontinuing the herb, symptoms appeared. He also mentioned regular weekly use of 40-50 cc alcohol. On presentation, hemodynamics was stable. He had conjunctival pallor and mild epigastric tenderness. The remainder of the physical examination was unremarkable. Laboratory results are shown in Table 1.

**Table 1:** Laboratory results

	**1**^st^ **day of admission**	**2**^nd^ **day of admission**	**8 months later**
WBC	18 600	10 500	7500
BUN	77.9	28	16
Cr	1.58	1.18	1.3
Hb	6.7	8.8	15.4
Hct	20.2	27.7	45
Plt	400 000	273 400	192 000

Abbreviations: WBC, White blood cell; BUN, blood urea nitrogen; Cr, creatinine; Hb, hemoglobin; Hct, hematocrit, Plt, Platelet.

**Figure 1:**
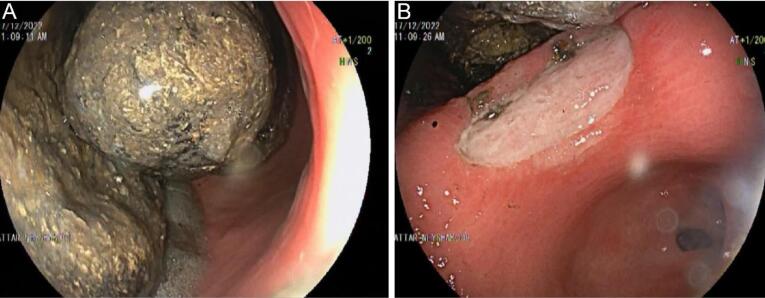
Endoscopic view of gastric phytobezoar and adjacent clean base ulcer at presentation (A, B)

 He first was hydrated and took intravenous pantoprazole. After 24 hours, an endoscopy was done for him, which showed a large green bezoar in the gastric body and an adjacent 7*20 mm clean base ulcer (Figure 1A, B). Multiple attempts were made to split the bezoar by snare but were not successful. Then, 100 cc cola was sprayed over the bezoar to dissolve it, but again, it was not effective. A biopsy from the ulcer margin was taken, and no evidence of malignancy or helicobacter pylori infection was found.

**Figure 2:**
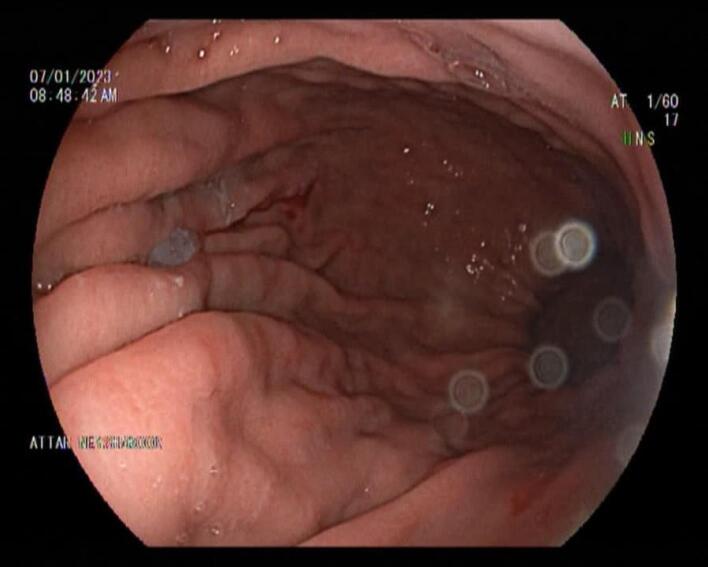
Endoscopic view after consumption of 1 week of Coca-Cola

 The patient was discharged on the 4th day of admission with oral proton pump inhibitor (PPI). After 2 weeks of using PPI, the patient was recommended to drink Zero Coca-Cola 500^cc^ three times per day for a period of one week. Endoscopy after 1 week showed that phytobezoar was completely resolved (Figure 2).

**Figure 3:**
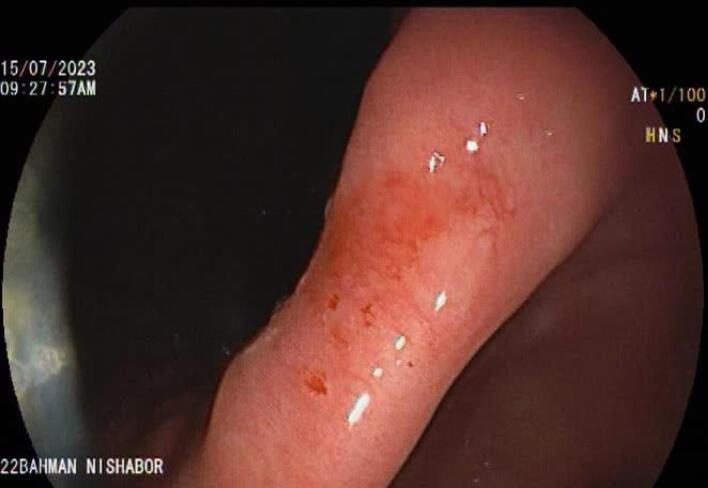
Surveillance endoscopy after 6 months revealed a completely healed ulcer

 The patient was recommended to continue oral PPI and repeat endoscopy after 3 months of PPI therapy. After 6 months (the patient returned to the clinic 3 months late), in surveillance endoscopy, the clean base gastric ulcer was healed completely, and only erythema was seen at the site of the previous ulcer, which was biopsied (Figure 3). The pathology result again had no evidence of malignancy or helicobacter pylori infection.

## Discussion

 In this report, we presented an elderly man with gastric phytobezoar for a long period. The phytobezoar led to a gastric ulcer, and the patient presented to ED with hematemesis. We treated the patient successfully with PPI and Coca-Cola, and surveillance endoscopy showed a completely healed ulcer.

 Bezoars are made from saliva and aggregation of indigestible materials. Bezoars can be classified into five main types based on their components: phytobezoars with the accumulation of vegetable and fruit fibers, trichobezoars with accumulation of hair, lactobezoars with milk accumulation, pharmacobezoars with the accumulation of medications and unusual bezoars that originate from paper, sand, etc.^1^ Bezoars mostly are asymptomatic, but symptoms that may occur include epigastric discomfort, early satiety, hematemesis (happened in this case), bloating, vomiting, and weight loss due to dysphagia and anorexia.^2^

 Phytobezoars are the most common type of bezoars that originated from indigestible cellulose, tannin, and lignin.^3^ There are different foods that can be the cause of phytobezoar, substances with great amounts of cellulose like prunes, raisins, celery, leeks, pumpkin, green beans, and substances with great amounts of lignin for example, flax seeds, root vegetables, wheat bran, edible vegetable and fruit seeds, peas, and peaches.^4^

 There are several ways to treat phytobezoars, including pharmacological dissolution with enzymes like cellulase,^5-7^ endoscopic procedures, and surgery. There are also several reports on using Coca-Cola for dissolving phytobezoar.^6,8-11^ Quantity, times, and duration of Coca-Cola usage are different in studies. The amount is between 500 mL-3000 mL daily for 24 hours to 6 weeks.^12^

 Although Fanta is more acidic than Coca-Cola Zero,^13^ it was not used to dissolve phytobezoars. This could be due to the corrosive nature of phosphoric acid^14^ in Coca-Cola instead of citric acid in Fanta. However, its dissolving property can also be aggravated by NaHco3, which exists in carbonated water and is present in both Coca-Cola and Fanta.^15^

 There are some case reports and one case series of intestinal and gastric phytobezoar from Iran.^16-18^ However, to the best of our knowledge, this is the first report of phytobezoar from Iran, which was treated with Coca-Cola.

## Conclusion

 This report also showed that in a patient with phytobezoar and gastric ulcer, Coca-Cola treatment can be safe and helpful. However, we must pay attention to prescribing the lowest possible amount of Coca-Cola or other acidic drinks to prevent further complications.
